# Magnetic Signatures and Magnetization Mechanisms for Grinding Burns Detection and Evaluation

**DOI:** 10.3390/s23104955

**Published:** 2023-05-22

**Authors:** Benjamin Ducharne, Gael Sebald, Hélène Petitpré, Hicham Lberni, Eric Wasniewski, Fan Zhang

**Affiliations:** 1 ELyTMaX IRL3757, CNRS, Univ Lyon, INSA Lyon, Centrale Lyon, Université Claude Bernard Lyon 1, Tohoku University, Sendai 980-8577, Japan; gael.sebald@insa-lyon.fr; 2 CETIM, 52 Avenue Félix Louat, 60300 Senlis, France; helene.petitpre@cetim.fr (H.P.); ext.hicham.lberni@cetim.fr (H.L.); eric.wasniewski@cetim.fr (E.W.); fan.zhang@cetim.fr (F.Z.)

**Keywords:** magnetic Barkhausen noise, magnetic incremental permeability, domain wall bulging

## Abstract

Grinding thermal damages, commonly called grinding burns occur when the grinding energy generates too much heat. Grinding burns modify the local hardness and can be a source of internal stress. Grinding burns will shorten the fatigue life of steel components and lead to severe failures. A typical way to detect grinding burns is the so-called nital etching method. This chemical technique is efficient but polluting. Methods based on the magnetization mechanisms are the alternative studied in this work. For this, two sets of structural steel specimens (18NiCr5-4 and X38Cr-Mo16-Tr) were metallurgically treated to induce increasing grinding burn levels. Hardness and surface stress pre-characterizations provided the study with mechanical data. Then, multiple magnetic responses (magnetic incremental permeability, magnetic Barkhausen noise, magnetic needle probe, etc.) were measured to establish the correlations between the magnetization mechanisms, the mechanical properties, and the grinding burn level. Owing to the experimental conditions and ratios between standard deviation and average values, mechanisms linked to the domain wall motions appear to be the most reliable. Coercivity obtained from the Barkhausen noise, or magnetic incremental permeability measurements, was revealed as the most correlated indicator (especially when the very strongly burned specimens were removed from the tested specimens list). Grinding burns, surface stress, and hardness were found to be weakly correlated. Thus, microstructural properties (dislocations, etc.) are suspected to be preponderant in the correlation with the magnetization mechanisms.

## 1. Introduction

High-performance mechanical components constitute critical parts in domains as diverse as transportation or energy production. Those elements (gears, bearings, camshafts, etc.) are made from expensive steel and must be ground after hardening to reach the required tolerances and surface qualities [1].

This machining process is complex, and for multiple reasons (unadapted cooling, excessive removal rates, or tool wear), it easily results in undesired outcomes (including reduced hardness or unexpected re-hardening) [2].

All these metallurgical flaws are commonly regrouped under the term “Grinding Burns” (GBs). GBs occur when the grinding energy generates too much heat, overcoming threshold levels and causing microstructural changes [3,4]. GBs modify the local hardness and can be a source of internal stress. GBs will shorten the fatigue life of critical, dynamically loaded components and can lead to severe failures.

Different methods exist for GBs detection [5]. A classic way is the so-called nital etching method which exposes the surface to be controlled to an etching process and reveals dark spots where the tested specimen is burnt [6]. This method is efficient but cannot be fully automatized. It requires skilled and qualified staff and involves polluting chemicals incompatible with modern industry’s green transition.

Micro-hardness characterization [7] and micro-structural images [8,9] are other solutions, but these methods are expensive, destructive, and impossible to implement for fast controls in a production line.

In addition to excellent mechanical behavior, steel components also share the common property of being ferromagnetic. The magnetic response of a ferromagnetic material is specific and comes from complex mechanisms interfering between space and time scales [10]. It depends on material properties, such as the composition, the microstructure, and the internal mechanical stress distribution. It also depends on external factors, such as the temperature or applied mechanical and magnetic stimulations [10,11].

The high sensitivity of the magnetic response to local structural variations makes the magnetization process examination an ideal candidate for the non-destructive detection of GBs. Nondestructive controls based on this principle have been exploited for years. Those methods are cheap, non-polluting, and can easily be set to perform reproducible tests on production lines. Different ways exist, but the most popular ones are based on the so-called Magnetic Barkhausen Noise (MBN) analysis [12,13,14]. A set of industrial equipment, such as the popular Stresstech^®^ controller, based on this peculiar magnetic manifestation has already been developed [15]. A significant problem for this device and, more generally, for the whole MBN analysis comes from the quasi-impossibility of distinguishing the effect of GBs from other influent factors (internal stress, dislocations, grain size, texture, plastic strain, precipitates, phase changes, impurities, etc.). This statement is even more true, considering that GBs act on these factors. In [16], this issue is solved using multiparameter Barkhausen noise measurements and collecting enough BN data from the thermally damaged zones to define a highly correlated combination of parameters and ideal experimental configurations.

The micromagnetic, multi-parametric, microstructure, and stress analysis 3MA^®^ developed by IZFP Fraunhofer Institute is an attractive alternative [17]. Similar to [16], 3MA accumulates and combines data from different magnetization responses and identifies the ultimate magnetic combination of indicators for GBs detection. Thus, 3MA is pragmatic and efficient but needs time-consuming experimental campaigns, leading to non-transposable results. As denoted by Withers et al. [18], Non-Destructive Testing (NDT) magnetic controllers are “mature, but a unified theory relating magnetic signals to basic magnetic parameters is lacking. At present signals are equipment supplier-specific”.

The configuration stage of industrial equipment always follows the same scheme and implies setting rejection thresholds from well-known specimens, pre-characterized with destructive and/or polluting methods. This method works but is expensive and time-consuming. It also restrains the controller exploitation to bounded experimental conditions. Finally, a slight change completely drops the method’s efficiency and reliability. As noted by Dobmann in [19], “However, besides the success story, we can also find critical remarks from industrial users. These are mainly to the calibration efforts and problems of recalibration if a sensor has to be changed because of damage by wear. Therefore, actual emphasis of R&D is to generalize calibration procedures”.

In this work, we opted for a different approach. In industrial equipment, correlation identification is based on data processing associated with a given experimental situation. No physical interpretation is associated with the tested indicator or the resulting correlated combination. Sometimes the magnetization mechanisms are triggered simultaneously, and their answers overlap, leading to even more complex interpretations. Still, a specific sensitivity characterizes every magnetization mechanism [20,21]. An ideal way to monitor GBs is to develop an experimental situation where the most responsive magnetization mechanism can be isolated and easily monitored. By focusing on the magnetization mechanisms instead of unrelated experimental observations, we hope to solve the reproducibility issue and converge toward magnetic indicators less dependent on the testing conditions.

A non-exhaustive list of the main magnetization mechanisms has been established to validate this statement. Then, specific experimental sequences and indicators were described and run for each mechanism. Linear correlations have been proposed using Pearson coefficients. Analyses have been provided along with the testing process and conclusions drawn regarding the ideal exploitation of the magnetization signature for grinding burns detection.

The manuscript is organized as follows:

- The tested specimens are described in the Section 2. This description includes the hardness and internal stress characterizations performed before the magnetic tests.
- A non-exhaustive list of the magnetization mechanisms is provided in the Section 3. Each mechanism is associated with a specific experimental situation and given indicators.
- Then, correlations are established. Together with discussions and conclusions, they constitute the Section 5 of this manuscript.

## 2. Tested Specimens

Two series of specimens have been studied in this work. The first series was made of X38CrMo16-TR martensitic stainless steel. The other was low carbon steel, 2 mm case-hardened, 18NiCr5-4.

**Figure 1:**
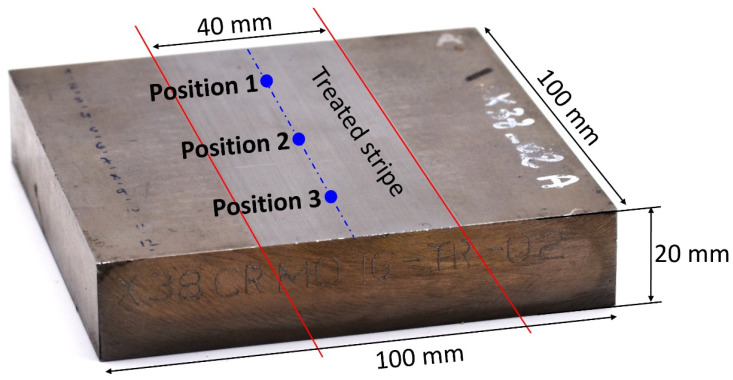
Tested specimen, dimensions, and identification of the treated stripe.

Furthermore, 100 × 100 × 20 mm^3^ rectangular pads (Figure 1) were prepared, including a central surface strip treated explicitly to exhibit five different grinding burn levels (from conform state to very strong burn).

**Figure 2:**
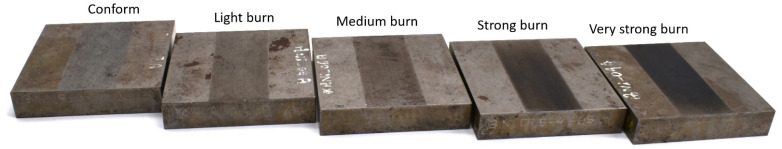
Illustration for the five grinding burn levels.

The light, medium, and strong burn levels (Figure 2) were obtained from over tempering. The very strong burn level from a complete strong tempering and re-hardening stage.

Before magnetic tests, hardness and internal stress characterizations were carried out on all specimens.

**Figure 3:**
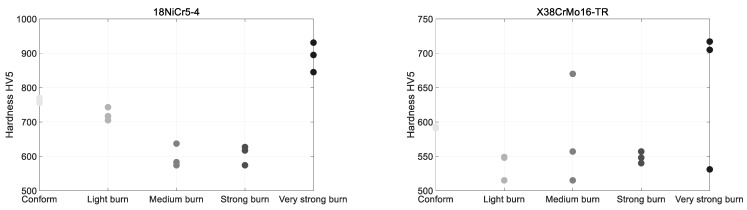
Hardness tests for both series of specimens.

Vicker hardness tests were performed with a 5 kg load on a DIATESTOR 2Rc series 7381 (Buehler, Lake Bluff, IL, USA). Figure 3 gives the resulting data. Each stripe was tested on three different positions (Figure 1) for reproducibility. The softening effect of the over-tempering is worth noting, especially true for the 18NiCr5-4. Oppositely, the complete re-hardening (very strong burn level) induced a significant superficial hardening effect on the specimen. Conclusions are less evident for the X38CrMo16-TR, where the relation between hardness and the surface treatment remains unclear.

**Figure 4:**
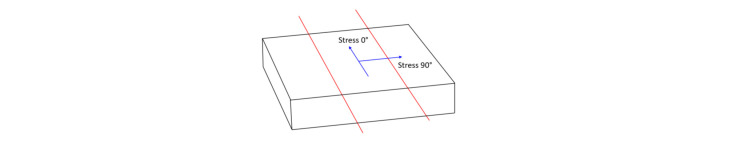

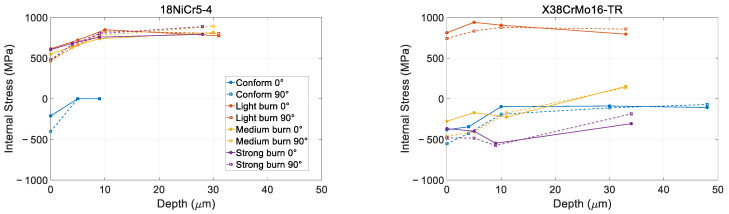
Internal stress trajectory.

X-ray diffraction stress measurements were completed in two directions (0 and 90°, as described in Figure 4 top illustration). Figure 4 bottom left and right charts give the stress profile for the 18NiCr5-4 and X38CoMo16-TR, respectively. Table 1 provides the superficial (upper layer) stress for all the specimens.

## 3. The Magnetization Mechanisms: Definition

Ferromagnetism arises from atomic magnetic moments of electronic origin becoming ordered into small regions known as magnetic domains. Each magnetic domain typically comprises 10^12^ to 10^18^ magnetic moments aligned in the same direction and orientation. At the domain boundaries known as domain walls, a change in the direction of the atomic magnetic moment progressively takes place over several hundred atoms (the exact number depends on energetical balance) [22].

**Figure 5:**
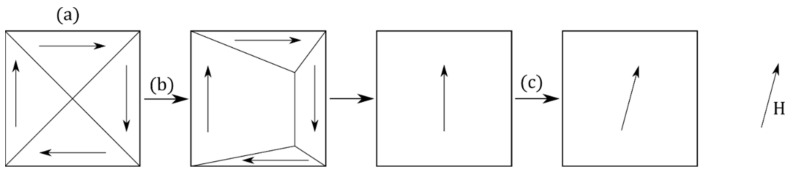
Schematic illustration of the magnetization process. (a) Demagnetized state, (b) Domain wall motion, (c) Magnetization rotation. In practice, the two mechanisms can coincide.

A ferromagnetic material’s magnetization process (Figure 5) supports multiple mechanisms: first, the magnetic domains with a magnetization oriented favorably to the applied magnetic field grow, while the domains unfavorably oriented decline in proportion. Then, the magnetization of the resulting domain, initially oriented along an easy axis, coherently rotates toward the direction of the applied magnetic field.

A large proportion of the magnetization mechanisms are associated with the magnetic domains and their distribution. These mechanisms include:

- The **domain wall bulging** mechanism is a local distortion of a domain wall under the influence of a low amplitude excitation H [23,24]. The so-called Magnetic Incremental Permeability (MIP) is the best way to characterize this mechanism. MIP is defined as the magnetic response to a steady, high amplitude quasi-static magnetic field (<1 Hz, max (H) > 5·H_c_) superimposed to a small amplitude alternative magnetic excitation (>50 kHz, H > H_c_/2, where H_c_ denotes the coercivity) [25]. The mathematical expression of MIP, μ_MIP_ is:
  (1) $$\mu_{MIP}=\frac{1}{\mu_{0}}\cdot \frac{∆B}{\Delta H}$$

**Figure 6:**
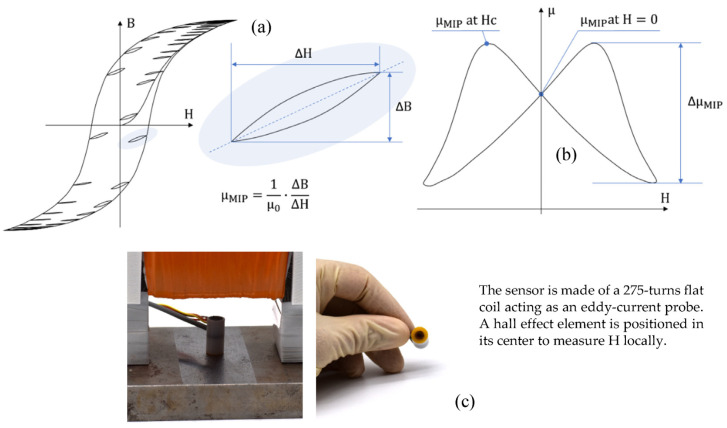
MIP illustration (**a**), graphical description of the domain wall bulging indicators (**b**), and pictures of the experimental setup and sensor (**c**).

The butterfly loop (Figure 6, left-hand side) is the usual magnetic signature associated with MIP. Δμ_MIP_, μ_MIP_ at H_c,_ and μ_MIP_ at H = 0 read on the butterfly loop are the MIP indicators we opted for in this study. MIP experimental setups give electrical signals; in this work, we used the semi-analytical process described in [26] to return permeabilities.

**Figure 7:**
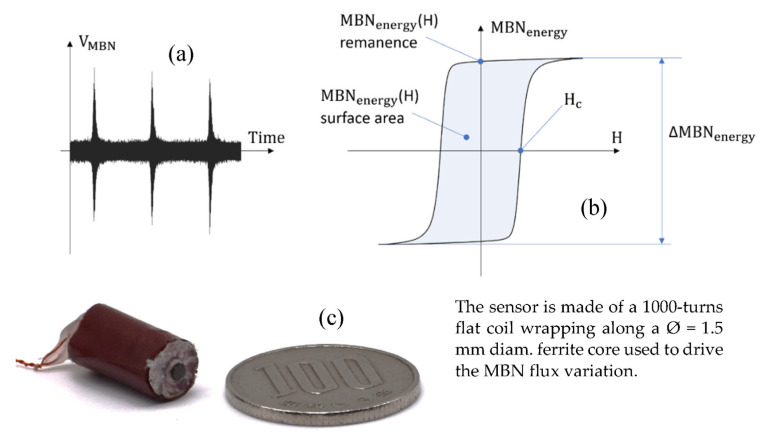
MBN typical signal (**a**), graphical description of the domain wall’s irreversible indicators (**b**), and pictures of the experimental sensor (**c**).

- The **domain wall’s irreversible motions** mechanism is associated with the domain walls breaking away from pinning sites under the influence of magnetic excitation. The ideal way to observe this mechanism is through the so-called Magnetic Barkhausen Noise (MBN) technique [27]. Domain wall motions generate local flux variations that trigger discontinuous magnetic flux density displayed as a series of electrical pulses induced in an inductive magnetic sensor [28]. The domain number is vast, the wall motions can be assimilated to a stochastic process, and the MBN raw signal is erratic and not reproducible. For repeatable results, time average indicators are always preferred for the MBN analysis, including the Magnetic Barkhausen Noise energy (MBN_energy_) described below [21,22,29]:

(2) $${MBN}_{energy}\left(t\right)=\int_{0}^{t}sign\left[\frac{dH}{dt}\left(s\right)\right]V_{MBN}^{2}\left(s\right)ds$$

where V_MBN_ is the sensor coil electromotive force. MBN_energy_ is not, strictly speaking, energy. It is more of an image of the kinetic energy associated with the domain wall motions [21]. Plotted as a function of H, MBN_energy_ leads to a hysteresis cycle characteristic of this mechanism (Figure 7). ΔMBN_energy_, H_c,_ remanence, and surface area read on the MBN_energy_(H) hysteresis loop are the MBN indicators that have been studied (Table 2).

**Figure 8:**
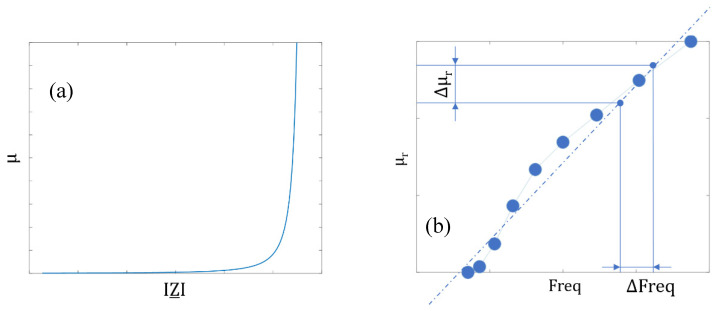
μ($\left|\underline{Z}\right|$) as obtained for a pancake coil with the D&D analytical expression (**a**), and example of μ_r_(Freq) once the conversion μ($\left|\underline{Z}\right|$) is applied (**b**).

- The **domain wall dynamic answer** (frequency dependence, ripples, and avalanches) is probably more a manifestation than a proper mechanism. In the well-known Bertotti’s Statistical Theory of Losses (STL), this behavior is associated with the excess losses W_exc_. It corresponds to the excess energy required by a dynamic magnetization process [30]. It is impossible to evaluate W_exc_ in NDT conditions with local surface measurements and magnetization waveforms far from the sinus shape imposed by the characterization standards. Instead, we opted for the frequency dependency of μ_MIP_ at H_c_ and μ_MIP_ at H = 0 as obtained with a frequency sweep of the MIP alternative contribution (see Figure 8a for illustration). MIP experimental setups provide electrical quantities (Z: the pancake coil complex impedance). We opted for the Dodd and Deeds (D&D) analytical method to convert Z into permeabilities (Figure 8b, [31]). Since this conversion process considers the eddy current contribution, the frequency dependence of the resulting permeability only stands on the domain wall dynamics. Figure 8b depicts the frequency dependence of μ_MIP_ at H = 0. This curve can be assimilated to a straight line. The slope of this line is the indicator we used.

**Figure 9:**
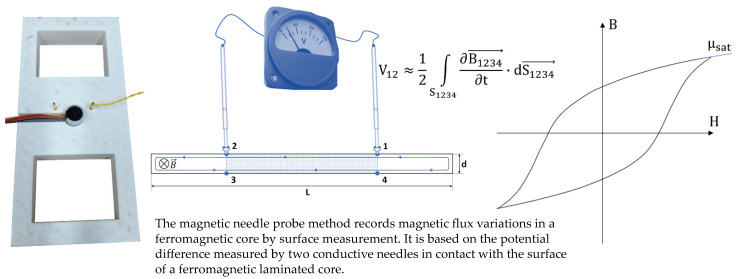
The point probe method [36]: experimental picture (**a**), 2D illustration and equation (**b**), graphical description of μ_sat_ the magnetization rotation indicator (**c**).

**Figure 10:**
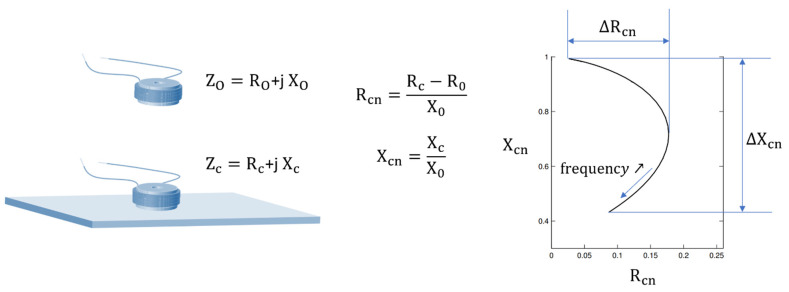
Macroscopic eddy current characterization and observation from indicators read on the classical ECT complex plan.

The remaining mechanisms are independent of the magnetic domain structure. These mechanisms include:

- The **magnetization rotation** mechanism is associated with the rotation of the magnetic moments under the influence of very high excitation. This mechanism starts once the saturation elbow is reached and continues up to full saturation. This mechanism can be characterized experimentally when a tested specimen is excited with a high amplitude rotating magnetic field [32,33]. Another method relies on unidirectional excitation and the study of the permeability at a very high saturation level when the single-domain state is reached. Here, magnetization variations are solely dependent on the magnetization rotation (Figure 9). In this study, surface B(H) hysteresis cycles were plotted. The pseudo induction B was obtained using the Magnetic Needle Probe (MNP) method [34,35]. The tangent surface H was measured with a Hall effect sensor (please note that it was also the case for the previous mechanisms). μ_sat_, the resulting permeability at maximal H, was used as a magnetization rotation indicator.

- The **macroscopic eddy currents** are also probably more a manifestation than a proper mechanism. This magnetization behavior is well known by the NDT community as it constitutes the basis of the Eddy Current Testing (ECT) method [37]. It is observable through the classical losses W_clas_ term in STL [30]. Eddy currents are frequency dependent and are generated whatever the amplitude of the magnetic excitation. They are not limited to ferromagnetic materials and will develop in every conductive material. The skin effect is a direct consequence of this mechanism [38]. It reduces the volume of the magnetized matter as the frequency increases. A classical approach with a pancake coil and indicators read on the complex impedance plane (Figure 10) has been used in this study to test this mechanism.

Table 2 combines all the tested indicators, such as their related magnetization mechanism and experimental setup.

## 4. Experimental Results and Correlation Analysis

### 4.1. Experimental Results

**Figure 11:**
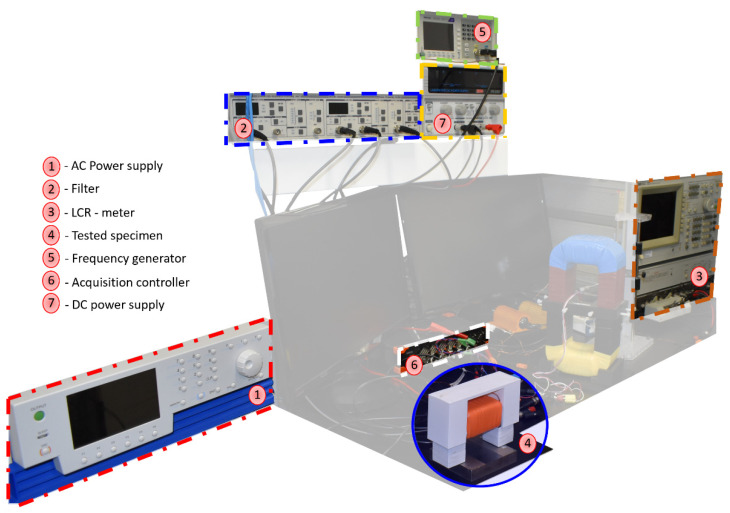
Overview of the experimental setup.

Table 3 displays the experimental data obtained for all tested specimens and indicators. It is worth mentioning that every ECT measurement has been completed in an unmagnetized state. For this, we performed a demagnetization process based on the slow decrease in an alternating magnetic excitation strength [39]. Figure 11 gives an overview of the experimental setup we have been using in this study.

**Figure 12:**
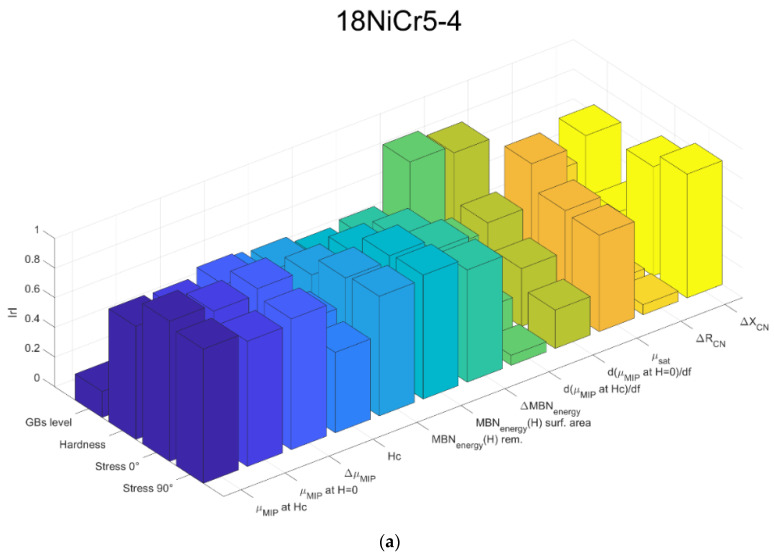

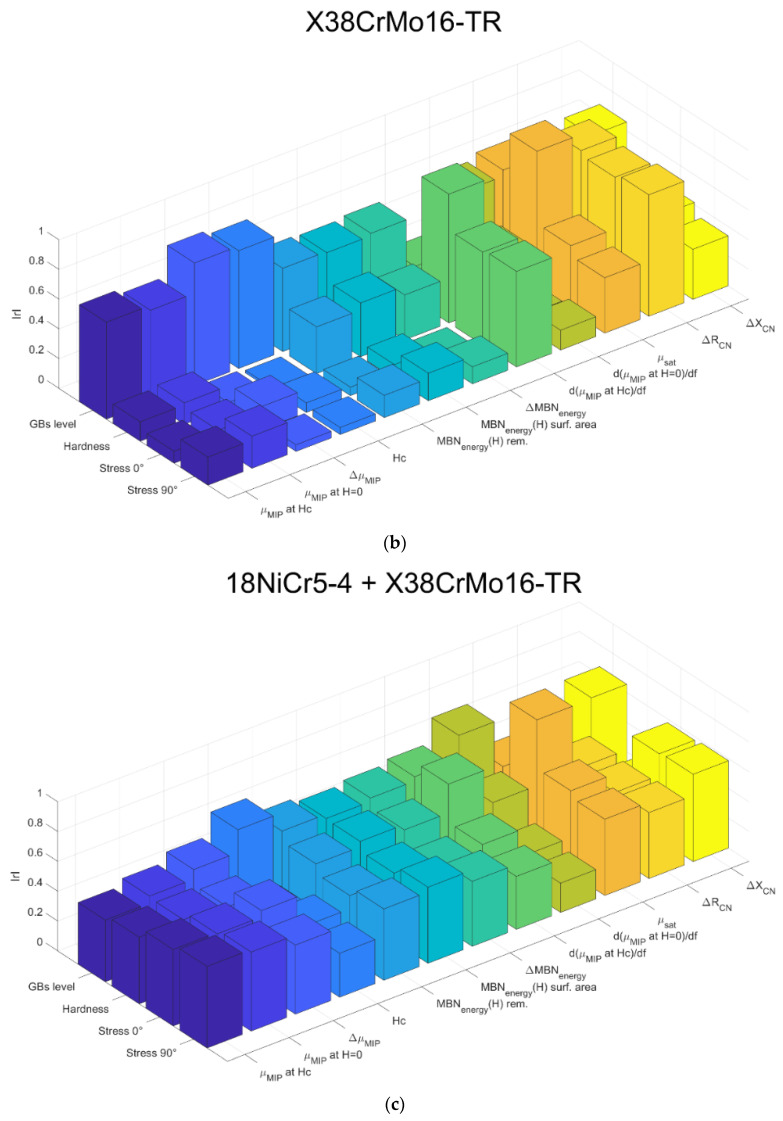
Mechanical/magnetic Pearson linear correlation coefficients for the 18NICr5-4 (**a**), X38CrMo16-TR (**b**), 18NICr5-4 + X38CrMo16-TR (**c**).

The next step in the analysis consists of computing the linear Pearson correlation factors ($\left|r\right|$). Figure 12 gives the resulting coefficients for both materials separately (Figure 12a,b) and combined (Figure 12c).

### 4.2. Analysis and Discussion

The ratio between average values and standard deviations in Table 3 gives an image of the magnetic indicators’ consistency. MIP indicators happened to be the most reliable. On the other side, the viability of μ_sat_ is much more questionable.

The more complex the experimental setup is, the less trustworthy the magnetic indicator becomes. Similarly, ECT results associated with LCR-meter measurements are very consistent; in contrast, indicators related to the domain wall dynamic answer obtained through the D&D analytical conversion are much more uncertain.

**Figure 13:**
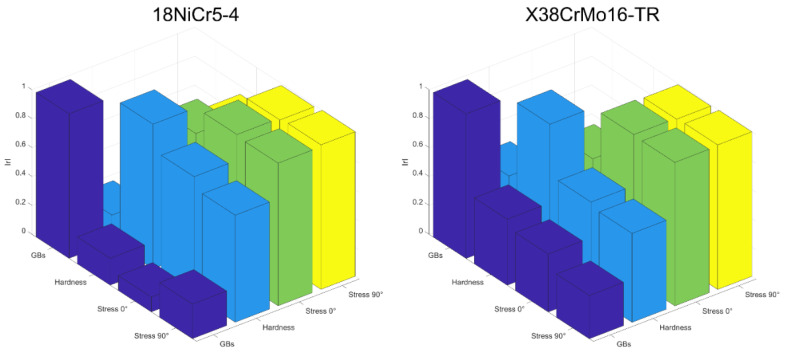
Pearson correlation coefficients for the mechanical pre-characterizations.

The overall correlation result (Figure 12c) is relatively weak. This observation could have been forecast by considering the significant differences between the materials’ mechanical properties and the quasi-absence of connection between them (especially true when the very strong burn specimens are considered). Figure 13 below confirms this statement by depicting the correlation properties between the mechanical pre-characterizations.

Thus, 0° and 90° stresses are the only correlated properties. Surprisingly, for both materials tested, GBs levels show very low linear correlations with the mechanical pre-characterizations. The fact that GBs act on the specimens’ mechanical properties is not questionable; it has constantly been mentioned in the scientific literature [5,40,41]. Still, any linear relationship is impossible to set from our experimental mechanical data.

Opposite behaviors can be described from a joint observation of Figure 12a,b, especially true for the mechanisms associated with the domain wall kinetic (reversible and irreversible domain wall motions, etc.). The very low correlation coefficients obtained with the X38CrMo16-TR mechanical properties and the MBN measurements were unexpected.

The scan depths of the tested methods are essential information to be considered in interpreting the results. GBs are superficial and lead to a thickness of the degraded layer lower than 150 μm (depending on the temperature and exposure time [42]). Amongst the tested methods, MBN (*f*: 0–100 kHz), MIP configurated in the high-frequency ranges (*f* > 50 kHz), and ECT (*f*: 10–1500 kHz) are surface characterization methods (scanning depth δ < 100 μm for MBN as discussed in [43,44]; scanning depth < 100 μm for MIP and δ < 70 μm for ECT as calculated from the skin depth equation). MNP at 50 Hz gives large-thickness scans incompatible with surface characterizations (the skin depth equation resolution gives δ = 1 mm for MNP).

Removing indicators with a low reproducibility ratio and unadapted scanned thickness reduces the tested magnetization mechanisms to the domain wall motions.

Once this reduction is made and focusing solely on GBs level estimation, a quick glance at Figure 12c leads to the absence of evident correlation. With 0.75, d(μ_MIP_ at H_c_)/d*f* is the higher coefficient; it is followed by H_c_, which gives the best results among the MBN coefficients.

Furthermore, d(μ_MIP_ at H_c_)/d*f* was obtained by sweeping up the alternative contribution of MIP tests combined with D&D reconstructions for the permeabilities. This process is meticulous and time-consuming, and the resulting data were limited. No standard deviations were available, and the trust level was low.

Moreover, H_c_ observations sound much more reliable, plus correlations between H_c_ and GBs have already been observed in the literature ([16], the position of peaks in [13]). H_c_ is strongly associated with the irreversible magnetic domain wall motions, the leading cause of losses under low-frequency magnetization processes. MBN is thus an ideal way to observe H_c_.

**Figure 14:**
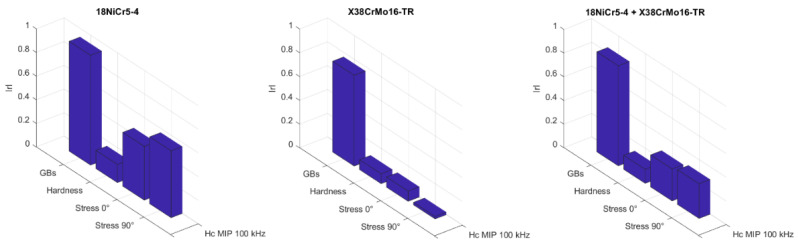
Pearson correlation coefficients for H_c_ read on the MIP response at 100 kHz.

However, H_c_ can also be observed through the peak position of the MIP signature. Additional MIP tests at higher alternative contribution (100 kHz) where the resolution is high, and the thickness of the scan layer more adapted were performed. Our objective is to confirm H_c_ and the irreversible domain motions as the best GBs indicator and magnetization mechanism. Results are depicted in Figure 14 below:

As expected, correlations are good, with almost 0.85 for both materials vs. GBs. Here, again, it is worth noting the weak correlation levels of the mechanical properties, confirming that other influent properties are dominant in the link with the magnetization processes. Microstructural characteristics like dislocation size and density are probably some of them. Finally, plotting the same correlations after removing the very strong burn specimens is interesting.

**Figure 15:**
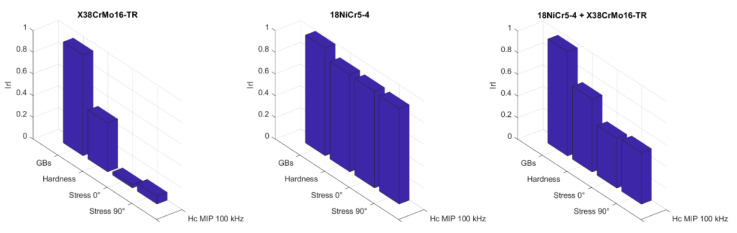
Pearson correlation coefficients for H_c_ read on the MIP signature at 100 kHz after removing the very strong burn specimen.

In that case, the Pearson coefficient correlating both materials and GBs reaches the outstanding 0.96. Many times in this study, we have noticed a change in tendency with the very strong burn specimen. As recalled in the Section 2 of this manuscript, a full re-hardening was completed to induce the very strong burn. The consequences of this intense metallurgical process on the microstructural properties and the magnetization process are considerable. Unfortunately, in terms of GBs detection, it significantly reduces the correlation properties. An evident difference between the correlation of the 18NiCr5-4 and the X38CrMo16-TR with the mechanical properties can also be observed in Figure 15. A correlation between the GBs level and these properties can only be established for the 18NICr5-4.

It is worth noting that the pre-characterizations carried out are local measurements (either on one point (stresses) or averaged on two or three points (surface hardnesses in Figure 3)). It can easily be accepted that the GBs state is not homogeneous over the entire surface and that a limited number of point measurements may not be rigorously representative. Therefore, if the magnetic measurements were not made at the same points, the established correlations may be slightly questioned (this is probably the case for the case-hardened martensitic stainless steel specimens). An appropriate averaging over the sample surface could be envisaged for a better estimation of the correlation properties.

## 5. Conclusions

In their survey of methods for GBs detection [5], He et al. limit the magnetic approaches to the MBN analysis. MBN is introduced as a promising nondestructive green technique but is limited to relative results that need to be compared with calibration blocks to design rejection thresholds. Even if not specified, this observation can be generalized to every industrial equipment based on magnetization signatures.

As recalled in the introduction of this study, the main issue faced by industrial magnetic controllers is related to the impossibility of discriminating the effect of GBs from other factors. To solve this issue, we adopted a new strategy focusing on the magnetization mechanisms. Where classical methods combining distinct measurements through complex mathematical formulas show a limited domain of validity, we expect to provide flexibility in the experimental conditions by concentrating on the adequate indicator and the most sensitive mechanism.

Based on this paradigm, we ran a complete study of GBs, the associated mechanical properties, and the magnetization mechanisms. Linear correlation coefficients were calculated to assess the relationship between all these experimental observations. A detailed analysis of these correlations leads to multiple conclusions summarized as follows:

- No correlation exists between the GB levels and the mechanical properties.
- H_c_ associated with the domain wall’s irreversible motions mechanism are, respectively, the most adapted indicator and magnetization mechanism.
- H_c_ read on the MIP butterfly loop measured in the high-frequency range of the alternative contribution is the best experimental situation; it reduces the scan thickness to the top layer where GBs are preponderant.
- The re-hardened “very strong burn” specimen shows an opposite trend and decreases the overall correlation coefficients.

The perspectives associated with this study are multiple. Of course, those first observations should be confirmed by additional experimental results (new specimens, new materials, etc.). A correlation survey with the microstructural properties would surely bring rich improvement to the current conclusions. Eventually, if H_c_ and high-frequency MIP characterization good results are corroborated, specific equipment should be designed to start exploiting them in the industrial context.

## Figures and Tables

**Table 1:** Superficial stress characterization.

	18NiCr5-4		X38CrMo16-TR	
	Stress 0° (MPa)	Stress 90° (MPa)	Stress 0° (MPa)	Stress 90° (MPa)
Conform	−210 ± 27	−401 ± 26	−381 ± 20	−553 ± 20
	−222 ± 26	−401 ± 27	−356 ± 19	−539 ± 20
Light burn	459 ± 21	401 ± 21	812 ± 18	741 ± 18
	614 ± 18	478 ± 17	752 ± 23	585 ± 25
Medium burn	549 ± 17	458 ± 17	−357 ± 41	−252 ± 38
	574 ± 17	478 ± 18	−276 ± 38	−464 ± 38
Strong burn	605 ± 17	479 ± 17	−367 ± 41	−481 ± 39
	649 ± 16	560 ± 16	−281 ± 39	−315 ± 40
Very strong burn	−85 ± 50	−178 ± 46	−415 ± 38	−438 ± 40
	−152 ± 50	−73 ± 51	−532 ± 41	−545 ± 39

**Table 2:** Compilation of all tested indicators combined with their magnetization mechanism and experimental method.

Magnetization Mechanism	Indicator	Unit	Experimental Method
Domain wall bulging	μ_MIP_ at Hc	H·m^−1^	MIP
	μ_MIP_ at H = 0	H·m^−1^	
	Δμ_MIP_	H·m^−1^	
Domain walls’ irreversible motion	Hc	A·m^−1^	MBN
	MBN_energy_(H) remanence	V^2^·s^−1^	
	MBN_energy_(H) surface area	A·V^2^·s^−1^·m^−1^	
	ΔMBN_energy_	V^2^·s^−1^	
Domain wall dynamic answer	d(μ_MIP_ at Hc)/d*f*	H·m^−1^·*f*^−1^	MIP
	d(μ_MIP_ at H = 0)/d*f*	H·m^−1^·*f*^−1^	
Magnetization rotation	μ_sat_	H·m^−1^	MNP
Macroscopic eddy current	ΔR_CN_	Ω	ECT
	ΔX_CN_	Ω	

**Table 3:** Compilation of all experimental results (average ± standard deviation).

			18NiCr5-4				
Magnetization Mechanism	Indicator	Unit	Initial State	Light Burn	Medium Burn	Strong Burn	Very Strong Burn
Domain wall bulging	μ_MIP_ at Hc	H·m^−1^	38.2 ± 0.24	41.4 ± 0.18	40.5 ± 0.8	40.6 ± 1	37.7 ± 0.35
	μ_MIP_ at H = 0	H·m^−1^	36.3 ± 0.63	38.7 ± 0.23	37.9 ± 0.45	37.8 ± 1.3	35.5 ± 0.74
	Δμ_MIP_	H·m^−1^	7.3 ± 1.33	9.1 ± 1.5	8.6 ± 2	8.8 ± 1.1	6.7 ± 1.34
Domain wall’s irreversible motion	Hc	A·m^−1^	1610 ± 138	1701 ± 13	1660 ± 60	1600 ± 50	1565 ± 84
	MBN_energy_(H) rem.	V^2^·s^−1^	11.37 ± 2.7	26.46 ± 7	34.71 ± 2	53.145 ± 6	24.86 ± 8
	MBN_energy_(H) surf. area	A·V^2^·s^−1^·m^−1^	96,345 ± 28,000	206,160 ± 55,300	245,940 ± 16,900	368,830 ± 43,000	174,390 ± 51,000
	ΔMBN_energy_	V^2^·s^−1^	37.9 ± 8.3	61 ± 9.5	82.5 ± 7.35	123.9 ± 12.5	61 ± 18
Domain wall dynamic answer	d(μ_MIP_ at Hc)/d*f*	H·m^−1^·*f*^−1^	8.45 × 10^−5^	6.73 × 10^−5^	3.12 × 10^−5^	2.67 × 10^−5^	−6.5 × 10^−6^
	d(μ_MIP_ at H = 0)/d*f*	H·m^−1^·*f*^−1^	5.47 × 10^−5^	6.73 × 10^−5^	4.5 × 10^−5^	2.96 × 10^−5^	−1.9 × 10^−5^
Magnetization rotation	μ_sat_	H·m^−1^	52.7 ± 3.7	50.3 ± 8.7	48.7 ± 1.3	37.5 ± 1.4	37.4 ± 6.6
Macroscopic eddy current	ΔR_CN_	Ω	0.2312	0.2441	0.2385	0.2164	0.2287
	ΔX_CN_	Ω	0.9317	0.9421	0.9415	0.9421	0.9409
			**X38CrMo16-TR**				
**Magnetization mechanism**	**Indicator**	**Unit**	**Initial state**	**Light burn**	**Medium burn**	**Strong burn**	**Very Strong burn**
Domain wall bulging	μ_MIP_ at Hc	H·m^−1^	42.15 ± 0.4	49.2 ± 1.5	52.5 ± 1.7	50.5 ± 0.43	49.6 ± 0.4
	μ_MIP_ at H = 0	H·m^−1^	40.9 ± 0.5	46.8 ± 1.35	49.5 ± 1.37	47.5 ± 0.47	46.85 ± 0.17
	Δμ_MIP_	H·m^−1^	4.13 ± 0.24	7.2 ± 0.7	9.6 ± 0.64	9.5 ± 0.19	9 ± 0.84
Domain wall’s irreversible motion	Hc	A·m^−1^	3650 ± 136	3190 ± 176	2930 ± 131	2960 ± 78	3020 ± 10
	MBN_energy_(H) rem.	V^2^·s^−1^	23.1 ± 0.6	56 ± 11.4	75.5 ± 9	72 ± 20	52 ± 10
	MBN_energy_(H) surf. area	A·V^2^·s^−1^·m^−1^	355,490 ± 13,500	724,615 ± 123,000	897,020 ± 113,000	899,575 ± 257,000	657,760 ± 121,000
	ΔMBN_energy_	V^2^·s^−1^	56 ± 2.7	115 ± 22	155 ± 16.4	149 ± 40	110 ± 19
Domain wall dynamic answer	d(μ_MIP_ at Hc)/d*f*	H·m^−1^·*f*^−1^	3.49 × 10^−5^	7.36 × 10^−5^	6.9 × 10^−5^	6.17 × 10^−5^	2.6 × 10^−6^
	d(μ_MIP_ at H = 0)/d*f*	H·m^−1^·*f*^−1^	4 × 10^−5^	1 × 10^−4^	6 × 10^−5^	1 × 10^−4^	−1.8 × 10^−5^
Magnetization rotation	μ_sat_	H·m^−1^	24.8 ± 1.14	21.6 ± 4.6	21.2 ± 3.5	24.1 ± 2.5	20.5 ± 1.4
Macroscopic eddy current	ΔR_CN_	Ω	0.1786	0.2242	0.1982	0.211	0.1842
	ΔX_CN_	Ω	0.9054	0.9134	0.9586	0.9654	0.9347

## Data Availability

Data available on request due to privacy/ethical restrictions.
